# Bone Mineral Density in Thalassemia Major Patients from Antalya, Turkey

**DOI:** 10.1155/2012/573298

**Published:** 2012-06-20

**Authors:** Ibrahim Aslan, Duran Canatan, Nihal Balta, Gulizar Kacar, Cengaver Dorak, Ahmet Ozsancak, Nurgul Oguz, Ruya Cosan

**Affiliations:** ^1^Department of Endocrinology and Metabolism, Antalya Education and Research Hospital, 07100 Antalya, Turkey; ^2^Hemoglobinopathy Diagnosis Center, Mediterranean Blood Diseases Foundation and Thalassemia Federation of Turkey, Antalya, Turkey; ^3^Antalya State Hospital, 07100 Antalya, Turkey; ^4^Department of Nuclear Medicine, Antalya Education and Research Hospital, 07100 Antalya, Turkey

## Abstract

*Aim*. We assessed the bone mineral density and related parameters in nine adults, thirty-eight pubertal, prepubertal totally forty-seven patients with thalassemia major living in Antalya, Turkey. *Materials and Methods*. We measured height and pubertal staging in last five years by six-month intervals. Average ferritin and hemoglobin concentrations were calculated for last three years. The levels of hydroxyproline, calcium, phosphorus, and creatinine were measured in 24 h urine, and those of parathormone, IGF 1, osteocalcine, alkaline phosphatase, calcium, ionized calcium, magnesium, phosphorus, creatine, blood glucose, thyroid stimulating hormone, alanine transaminase, and aspartate transaminase were determined in serum, and also the bone mineral density was measured. *Results*. The average L1–L4 bone mass density was 27.1 ± 10.1 g cm^−2^; the average bone mineral content was 0.65  ±  0.11 g. of the patients with a Z-score under 2.5. A moderate relationship was found between the bone mass density age and height. Subjects in low pubertal staging and short stature (<3% percentile) have significantly lower bone mass densities *P* < 0.001. *Conclusion*. he prevalence of osteoporosis is high in patients with thalassemia major, possibly related to delayed puberty.

## 1. Introduction

Patients with thalassemia major exhibit several endocrine disorders. A decrease of bone mineral density and early onset of osteoporosis have been reported in young patients [1–5]. Among the factors in thalassemia that contribute to development of osteoporosis are chronic hypoxia, medullar expansion, iron accumulation, desferrioxamine therapy, abnormal calcium-phosphorus balance, highbone turnover, and hormonal insufficiency [1, 6, 7]. Sex steroids play important roles in bone metabolism [8]. Delayed puberty and hypogonadism are important risk factors in the decline of bone mineral density (BMD) in thalassemia patients [8]. Male patients are more often and seriously affected than female patients [7]. The present study aims to evaluate osteoporosis and associated parameters in patients with thalassemia major living in Antalya, Turkey.

## 2. Materials and Methods 

### 2.1. Patients 

The study was conducted according to the ethical guidelines for human studies established by the Ethics Committee of the Antalya Education and Research Hospital. A total of 47 patients (38 prepubertal-pubertal, 9 adult) was included in the study. Pubertal development was evaluated according to Tanner's classification. We measured the height of thalassemic patients every six months for last 5 years.

Patients receiving blood transfusions every three weeks were given 40–50 mg/day desferrioxamine. Two male and two female patients had been taking Vitamin D and calcium supplements after osteoporosis diagnosis. 

### 2.2. Laboratory Procedures

The average ferritin and hemoglobin levels before and after transfusion were registered for the last three years. Fasting blood samples and 24-hours urine samples were obtained from all of the participants. The phosphorus, BUN, creatine, and other parameters were measured using an Olympus AU400 autoanalyzer and reagents (USA). The concentration of calcium ions (Ca^2+^) was measured by means of a Medica Prolyte Menarini Il-Lyte Spotlite Electrolyte Analyzer System. Serum magnesium (Mg^2+^) was measured in a Boehringer/Hitachi spectrometer using commercially available kits from Roche Diagnostics, Basel, Switzerland. 

The serum parathyroid hormone (PTH) values were obtained by the immunoradiometric (IRMA) assay using a minigamma counter. Active intact PTH was used as reference in all measurements.

The BMD was measured at the Department of Nuclear Medicine of Antalya State Hospital, using a Hologic 4500 Elite Full Body Bone Densitometer. Only the L1–L4 area was considered in evaluation of the data. The statistical treatment of the data was carried out using MS Excel computer program. 

## 3. Results

**Figure 1:**
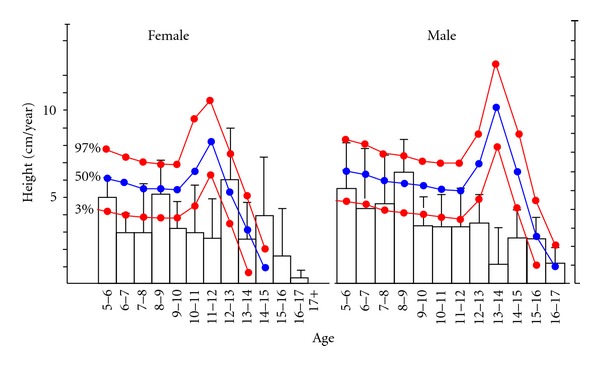
Average growth of thalassemic patients.

The height of 21 patients (8 females, 13 males) was under the 3% percentile of their age group. Pubertal growth of thalassemic patients is lower than Turkish children (Figure 1). Eighteen patients were at the P1 level of pubertal development, and 11 were at the P2 level. Three of the patients were diagnosed with hypothyroidism, one with hypoparathyroidism and three with diabetes mellitus. 

The Ca^2+^ level was low in 13 of the patients while 18 had low Mg^2+^ levels. The phosphorus level was high in 33 patients, and the PTH values were high in 37 patients. The average creatinine clearance (Ccr) values were calculated as 88 ± 33 ml/min. There were no relationships between these values and BMC or BMD. All averaged biochemical data were shown in Table 1.

**Figure 2:**
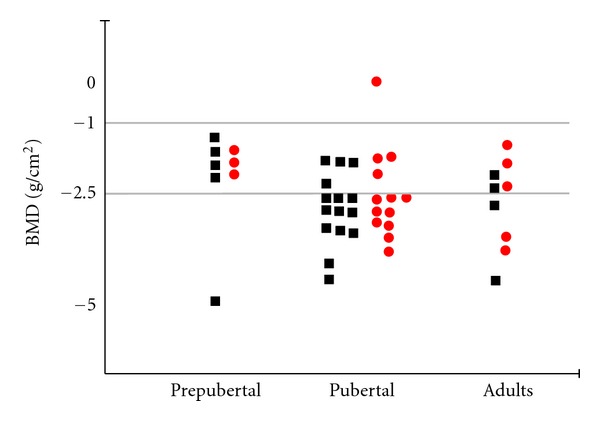
L1–L4 bone mass density (*Z* Score) in patients with thalassemia major.

**Figure 3:**
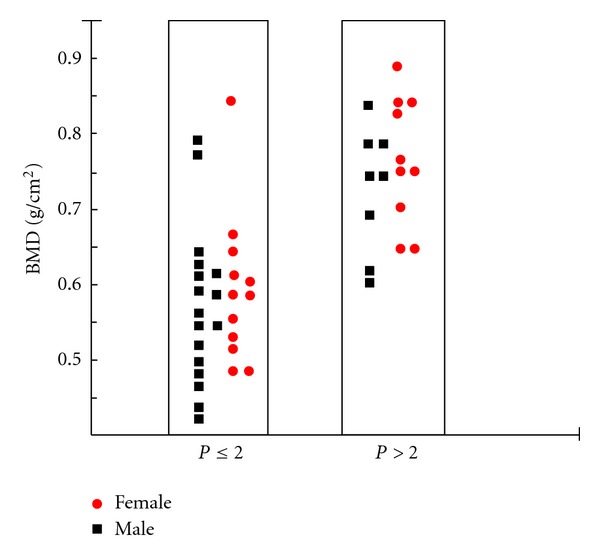
Puberty-bone mass density relationships in patients with thalassemia major.

As seen in Figure 2, the *Z* score was under −2.5 in 25 of the thalassemia patients. The average BMD (L1–L4) was 27.05 ± 10.5 g/cm^2^, and the average BMC was 0.645 ± 0.11 g. A moderate relationship was seen between age, height, and BMD. The BMD of patients whose height was under 3% percentile was statistically different in comparison with the rest of the subjects: 0.506 ± 0.097 g/cm^2^ for height under 3% percentile, 0.693 ± 0.111 g/cm^2^ for patients above that height level. The BMD levels were statistically different in patients whose pubertal stage was low in comparison with the others. The patients in ≤P2 development stage were 0.587 ± 0.09 g/cm^2^, and those above P2 were 0.739 ± 0.08 g/cm^2^, *P* < 0.001. The L1–L4 BMD values and pubertal level of the thalassemia patients can be seen in Figure 3.

## 4. Discussion 

Osteoporosis is commonly seen in young thalassemia patients [1–5]. The frequency of this condition can be as high as 51% [3], with a decline of over 30% of spinal mineral density in comparison to normal subjects [1]. In agreement with these reports, the majority of patients in this study were diagnosed with osteoporosis based on their L1–L4 vertebral bone mineral densities. 

There are several factors involved in the development of osteoporosis in thalassemia patients. Among them are chronic hypoxia, medullar expansion, and iron accumulation. In addition, the use of desferrioxamine results in abnormal calcium-phosphorus balance and hormonal disorders [1]. Some of these factors were evaluated in the present investigation. 

Low pre- and posttransfusion hemoglobin concentrations demonstrate that the patients included in this study suffered from chromic hypoxia, and their ferritin levels were elevated because of transfusions resulting in iron accumulation in many tissues [1, 9, 10]. 

We did not find elevated ALP and osteocalcin levels in the patients, suggesting reduced osteoblastic activity, possibly related to iron accumulation in osteoblasts. Iron accumulation in liver can also lead to defects in the synthesis of vitamin D [1]. 

In addition to these functional defects due to iron accumulation, frequent transfusions and use of desferrioxamine can lead to lower serum Ca^2+^, Mg^2+^ and higher *P* and PTH levels [1]. All of these factors also contribute to the development of osteoporosis.

Some endocrine complications including short stature, delayed puberty, hypogonadism, and diabetes mellitus can be seen in thalassemia patients [8, 11]. These are also important risk factors in the decline of BMD [1, 2, 5, 8, 11]. Diabetes mellitus, hypoparathyroidism, delayed puberty, and hypogonadism were seen among patients whose height is under the 3% percentile. 

All of the findings in this study showed that delayed puberty and hypogonadism are important risk factors in the decline of bone mineral density (BMD) in thalassemia patients. Male patients are more often and seriously affected than female patients.

Development of osteoporosis is high in thalassemic patients possibly related to delayed puberty. We must add the sex steroid replacement to thalassemic patients as a part of osteoporosis therapy.

## Figures and Tables

**Table 1:** Particulars of the model.

Parameter	Average ± SD
Ca^2+^ (mmol/L)	1.2 ± 0.1
Ca (mg/dL)	9.1 ± 0.6
P (mg/dL)	5.3 ± 1.7
Mg (mg/dL)	1.65 ± 0.2
PTH (pg/mL)	85 ± 44
Osteocalcin (ng/mL)	14.9 ± 9.7
IGF1 (ng/mL)	423 ± 276
ALP (UL/L)	1174 ± 69
ALT (U/L)	88.5 ± 78
AST (UL/L)	68.2 ± 46
Albumin (g/dL)	4.7 ± 0.2
Total protein (g/dL)	8 ± 0.8
Pretransfusion Hb (g/dL)	9.2 ± 0.7
Posttransfusion Hb (g/dL)	11.8 ± 0.8
Ferritin (ng/mL)	6594 ± 4428
Urine creatinine (mg/dL)	57 ± 20
Urine calcium (mg/dL)	6.6 ± 2.4
Urine phosphorus (mg/dL)	20.8 ± 2020.3 ± 9.5
